# A New Species of the Genus *Acrossocheilus* Oshima, 1919 (Cypriniformes: Cyprinidae) from the Dabie Mountains

**DOI:** 10.3390/ani15050734

**Published:** 2025-03-04

**Authors:** Tian-En Chen, Jia-Xin Xu, Peng-Ju Li, Huan-Fu Hu, Kai Gao, Hai-Peng Zhao

**Affiliations:** 1 School of Life Sciences, Henan University, Kaifeng 475004, Henan, China; 2 Henan Dabieshan National Field Observation and Research Station of Forest Ecosystem, Kaifeng 475004, Henan, China; 3 Xiaoqinling Ecological Restoration Field Observation and Research Station of the Yellow River Basin, Henan University, Kaifeng 475004, Henan, China; 4 Xinyang Academy of Ecological Research, Xinyang 464000, Henan, China; 5 Dabie Mountain National Nature Reserve of Henan, Xinyang 464236, Henan, China

**Keywords:** biodiversity, Cyprinidae, Henan, new species, taxonomy

## Abstract

*Acrossocheilus* Oshima, 1919, a cyprinid genus of Cyprinidae in southern China and currently comprises 26 valid species. In this study, we describe *Acrossocheilus dabieensis* **sp. nov.** from the Dabie Mountains, China. This new species differs from its congeners by the following combination of characters: The second primary vertical bar (PB2) is situated beneath the anterior origin of the dorsal fin in females and subadult males. Vertical bars extend to the end of the ventral abdomen in juveniles, and they gradually recede above the lateral line in adult females, whereas they are absent in adult males. The last unbranched dorsal-fin ray is slender with a smooth posterior margin. Phylogenetic analyses based on the mitochondrial DNA sequences indicate that *A. dabieensis* **sp. nov.** is a monophyletic group, and it forms a sister group with *A. kreyenbergii*, reinforcing the status of the new species. A key to the barred species of *Acrossocheilus* is also provided.

## Simple Summary

The family Cyprinidae Cuvier, 1817 includes more than 3000 species worldwide and has an important influence on the freshwater fishery economy. *Acrossocheilus* Oshima, 1919 is a group of small- to medium-sized barbine species, which are widely distributed in Laos, Vietnam and southern China. One new species, *Acrossocheilus dabieensis* **sp. nov.**, from the Dabie Mountains, Henan Province, China, is described and illustrated in this study.

## 1. Introduction

Cyprinidae Cuvier, 1817 is the most species-rich family of freshwater teleosts and comprises more than 3000 described species [1]. The cyprinid genus *Acrossocheilus* Oshima, 1919 (Cyprinidae: Barbinae) was erected with *Gymnostomus formosanus* Regan, 1908 as its type species, and it represents a group of small- to medium-sized barbine species [2]. The genus *Acrossocheilus* includes 26 known species, which are widely distributed in Laos, Vietnam and southern China [3,4,5]. Southern China is the main distribution area of this genus [3,6]. The fishes in the genus *Acrossocheilus* commonly inhabit the middle and lower reaches of swift streams or standing waters, with relatively large populations. As a group of omnivorous fish, they mainly feed on algae, aquatic arthropods, or other organic matter [7]. The adults are tender and delicious in taste, possessing highly polyunsaturated fatty acids [8]. The species of *Acrossocheilus* are a group of freshwater fish with great ecological and economic significance in Southeast Asia [2,9].

The genus *Acrossocheilus* can mainly be distinguished from other genera by body colors and stripes, the presence of a medially interrupted lower lip with two thick lateral lobes, and the absence of serrations at the posterior edge of the last unbranched dorsal fin [2,4,10]. The taxonomic study of *Acrossocheilus* remains a matter of debate as varying numbers of species or subspecies have been recognized within this genus [5]. Based on diverse body colorations, the species of *Acrossocheilus* can be categorized into two species-groups: a barred group and a non-barred group [5,11]. The former group contains 16 species characterized by five to eight vertical black bars on the flanks, including *A. beijiangensis*, *A. clivosius*, *A. fasciatus*, *A. hemispinus*, *A. iridescens*, *A. jishouensis*, *A. kreyenbergii*, *A. lamus*, *A. longipinnis*, *A. microstoma*, *A. monticola*, *A. paradoxus*, *A. parallens*, *A. spinifer*, *A. wenchowensis*, and *A. wuyiensis* [4]. The latter comprises ten species devoid of vertical black bars yet possessing a plain or indistinct black stripe along the lateral line, such as *A. aluoiensis*, *A. baolacensis*, *A. ikedai*, *A. macrophthalmus*, *A. malacopterus*, *A. multistriatus*, *A. rendahli*, *A. xamensis*, *A. yalyensis*, and *A. yunnanensis* [6]. Recent studies revealed that the barred group of *Acrossocheilus* is regarded as a polyphyletic group based on molecular phylogenetic analyses [4,5,6,9,10,11,12,13]. One study suggests that the barred group of *Acrossocheilus* consists of precisely 16 species [4].

The Dabie Mountains, situated at the boundary of Anhui, Henan, and Hubei Provinces, are renowned as a biodiversity hotspot due to their abundant animal and plant resources. This region has a warm and humid monsoon climate in the north subtropical zone and serves as the watershed between the Yangtze and Huai Rivers [14]. The species of *Acrossocheilus* was initially reported as *A. parallens* from the Dabie Mountains in Shangcheng County, Henan Province, and then was later documented as *A. hemispinus hemispinus* in “*The Fauna of Fishes from Henan*” [15,16,17]. However, certain taxonomic inquiries indicate that this species is neither *A. parallens* nor *A. hemispinus hemispinus*. The taxonomic status of this species still remains debatable [18,19]. According to previous taxonomic studies, *A. hemispinus* has long been confirmed to contain two subspecies: *A. h. hemispinus* from the Minjiang River in Fujian Province and the Qiangtang River in Zhejiang Province, and *A. h. cinctus* from the Pearl River in the Guangxi Autonomous Region [2,17,20,21,22]. Subsequently, in light of the differences in adult color pattern and the length of the longitudinal stripe, *A. h. hemispinus* and *A. h. cinctus* were proposed as two distinct species [12]. Furthermore, the valid status of *A. hemispinus* and *A. cinctus* (=*A. kreyenbergii*) was also supported by molecular phylogenetic analyses [23,24].

This study examined the specimens of *Acrossocheilus* collected from the Dabie Mountains. Based on the morphological and molecular phylogenetic evidence, this species is described as a new species herein. An updated diagnostic key to the barred species of *Acrossocheilus* is also provided.

## 2. Materials and Methods

### 2.1. Taxon Sampling

**Figure 1:**
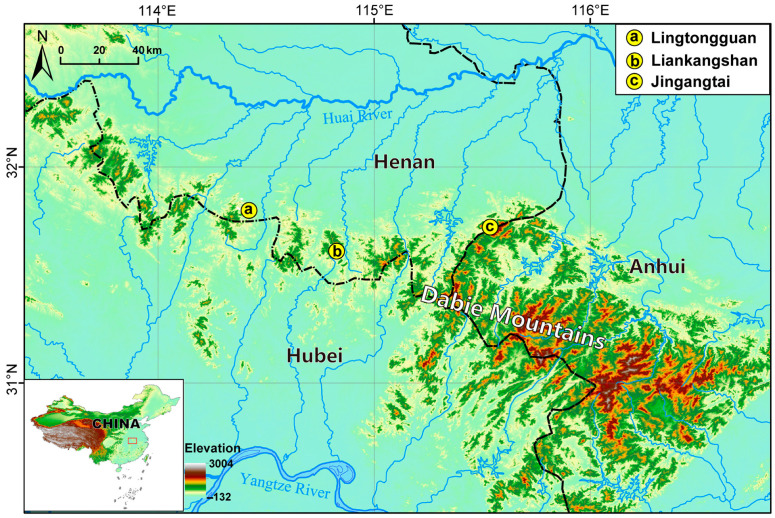
Topographic map showing the distribution of *Acrossocheilus dabieensis* **sp. nov.**

A total of 93 specimens of *A. dabieensis* was collected from three locations in the Dabie Mountains in China from 2017 to 2025 (Figure 1). All voucher specimens were deposited in the Biodiversity Museum, Henan University, Kaifeng, China (HENU) and the National Zoological Museum, Institute of Zoology, Chinese Academy of Sciences, Beijing, China (ASIZB). The right pectoral fins and associated muscle tissues were cut off and preserved in a 95% ethanol solution for DNA extraction. After the extraction of these tissue samples, the specimens were initially fixed in a 10% neutral formalin solution for one week, and then transferred to a 75% ethanol solution for long-term preservation.

### 2.2. Morphometrics

A total of 93 specimens of *A. dabieensis* were measured in this study. Measurements were conducted using digital calipers, recorded from point to point, and rounded to the nearest 0.1 mm. Counts and measurements were taken on the left side of individuals following the protocols established by Kottelat [11], including four supplementary measurements: predorsal length, prepectoral length, prepelvic length, and preanal length [5,12,13]. The abbreviations utilized in this study are as follows: HL for head length and SL for standard length.

Sexual determination is mainly based on tuberculation and body coloration. The snout tip and anterior portion of the lachrymal possess tubercles in adult males, but these tubercles are absent in adult females. Vertical bars are absent in adult males, whereas they are clearly defined and gradually recede above the lateral line in adult females. Compared with males, the body color and stripes of females remain relatively stable.

### 2.3. DNA Extraction, PCR Amplification, and Sequencing

Total genomic DNA was extracted from muscle samples preserved in 95% ethanol using the Vazyme Biotech Co., Ltd. (Nanjing, China). Whole Genome DNA Extraction Kit, following the manufacturer’s instructions. Standard polymerase chain reaction (PCR) methods were carried out in 25 μL reactions. The D-loop region of the mitochondrial DNA (mtDNA) spanning from the 5′ end of tRNA-Pro to the 3′ end of 12S rRNA was amplified via PCR [25,26]. The Primer sequences are Loop 1–2 (H) (5′-GCATC GGTCT TGTAA TCCGA-3′) and Loop 2–2 (L) (5′-GCCCG CTCCT CGTCT CCGGG-3′) [26,27]. The PCR program was set under the following conditions: 98 °C for 30 s pre-denaturation; 98 °C for 10 s of denaturation; 55 °C for 5 s annealing; 72 °C for 8 s extension; 35 cycles; 72 °C for 1 min extension; and 4 °C for holding. PCR products were detected through 1% agarose gel electrophoresis, and the bands were visualized under a long-wave UV lamp. The luminescent electrophoretic bands were isolated by cutting the gel. Finally, the PCR products were sent to the Beijing Genomics Institute (BGI) for sequencing in both directions.

The crude sequencing chromatograms were manually curated using SnapGene 6.0.2 [28] to eliminate any inaccurate sequencing regions. Only sequences with unambiguous chromatograms in both directions were regarded as reliable and were utilized in subsequent analyses. To determine the status of the new species, we retrieved 110 gene sequences representative of 14 species of the genus *Acrossocheilus* from GenBank (Table S1). Two species of Cyprinidae, *Onychostoma gerlachi* (Peters, 1981), and *O. barbatulum* (Pellerin, 1908), were used as outgroups for phylogenetic analyses.

### 2.4. Phylogenetic Analyses

All sequences were examined, compiled, and edited using both SnapGene 6.0.2 [28] and MEGA 11 [29]. Multiple sequence alignments were performed with the default parameter settings of MUSCLE in MEGA 11 [29]. All sequences were submitted to GenBank (for accession numbers see Table S1). To construct phylogenetic trees, Bayesian Inference (BI), Maximum Likelihood (ML), and Neighbor-Joining (NJ) analyses were performed based on the D-loop gene sequences.

BI analysis was performed using MrBayes 3.2.6 [30]. The best-fitting substitution models and partition scheme were estimated for the combined dataset by using PartitionFinder v.2.1.1 [31]. The best-fitting model was HKY + I + G under the Bayesian Information Criterion (BIC). Two independent runs with four Markov Chain Monte Carlo (MCMC) chains were performed for 2,000,000 generations with a sampling frequency of 100, until the mean standard deviation of the split frequencies was less than 0.01 [32]. ML analysis was performed with the software IQ-TREE 2.2.7 [33]. The best-fitting substitution model, HKY + F + I + R2, was selected via IQ-TREE 2.2.7 [33] under the BIC. The analysis involved 10 thorough bootstrap runs initiated at random, and 1000 bootstrap repetitions were performed to obtain bootstrap values (BV). NJ analysis was constructed with MEGA 11 [29] under the Kimura two-parameter (K2P) model with 1000 bootstraps.

## 3. Results

### 3.1. Taxonomic Account

**Figure 2:**
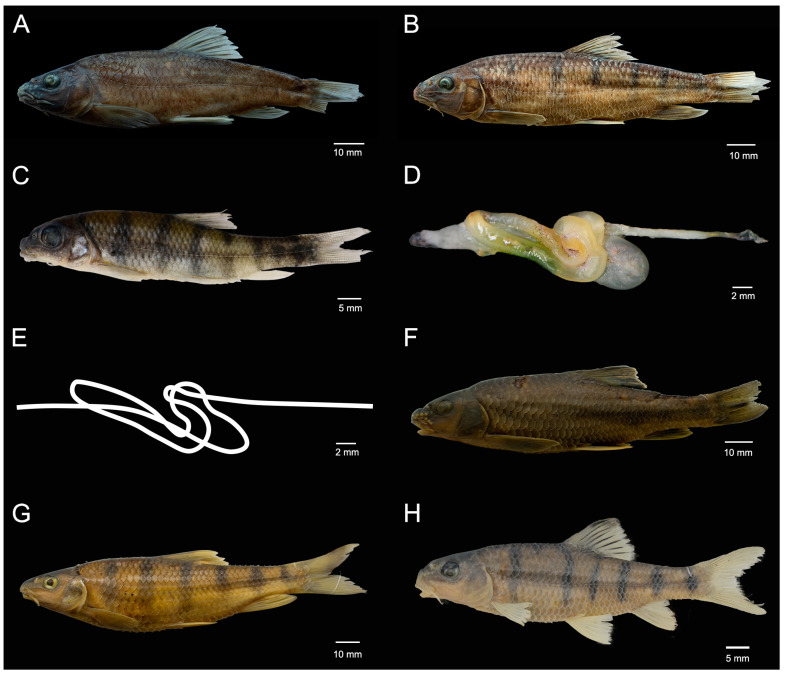
Comparisons of *Acrossocheilus dabieensis* **sp. nov.**, *Acrossocheilus kreyenbergii*, and *Acrossocheilus wuyiensis.* (**A**) Adult of *A. dabieensis* **sp. nov.**, lateral view (HENUJGT002); (**B**) female adult of *A. dabieensis* **sp. nov.**, lateral view (HENUJGT001); (**C**) juvenile of *A. dabieensis* **sp. nov.**, lateral view (HENULKS020); (**D**) intestine coiling of *A. dabieensis* **sp. nov.** (HENULKS043); (**E**) intestinal line drawing of *A. dabieensis* **sp. nov.** (HENULKS043); (**F**) male adult of *A. kreyenbergii*, lateral view (ASIZB223833); (**G**) female adult of *A. kreyenbergii*, lateral view (ASIZB231514); and (**H**) male adult of *A. wuyiensis*, lateral view (ASIZB227079).

***Acrossocheilus dabieensis*** **Chen & Zhao, sp. nov.**

urn:lsid: zoobank.org: act: 0AEDB47E-16B9-450B-9FD0-FB223E70CE40 (Figure 2A–E).

**Holotype.** This comprised HENUJGT002, a 100.2 mm SL, adult male, collected by Huan-Fu Hu in Jingangtai Nature Reserve (31°43′20″ N, 115°32′20″ E, 551 m a.s.l.), Shangcheng County, Xinyang City, Henan Province, China, 14 November 2017.

**Paratypes.** These comprised HENUJGT001, HENUJGT003–HENUJGT009, 52.1–125.1 mm SL, two males and five females and the same collection information as the holotype, as well as HENUJGT0010–HENUJGT0037, 36.4–124.6 mm SL, 8 males and 20 females; these were collected by Tian-En Chen, Peng-Ju Li, and Di Zhao in Jingangtai Nature Reserve (31°43′30″ N, 115°32′24″ E, 438 m a.s.l.), Shangcheng County, Xinyang City, Henan Province, China, 12 Jan. 2025. HENULKS001–HENULKS003, 33.9–76.3 mm SL, one male and two females were collected by Tian-En Chen, Kai Gao, and Hai-Peng Zhao in Lingtongguan (31°49′48″ N, 114°22′48″ E, 169 m a.s.l.), Luoshan County, Xinyang City, Henan Province, China, 20 October 2022. HENULKS004–HENULKS0012, 77.5–121.2 mm SL, one male and eight females were collected by Tian-En Chen, Kai Gao, and Hai-Peng Zhao in Liankangshan National Nature Reserve (31°36′37″ N, 114°49′41″ E, 370 m a.s.l.), Xinxian County, Xinyang City, Henan Province, China, 22 October 2022. HENULKS0013–HENULKS0056, 28.6–103.2 mm SL, 12 males and 31 females, were collected by Tian-En Chen, Peng-Ju Li, and Di Zhao in Lingtongguan (31°49′43″ N, 114°22′50″ E, 127 m a.s.l.), Luoshan County, Xinyang City, Henan Province, China, 10 January 2025.

**Diagnosis.** *Acrossocheilus dabieensis* **sp. nov.** can be distinguished from its congeners by the following morphological characteristics: (1) The second primary vertical bar (PB2) situated beneath the anterior origin of the dorsal-fin in females and subadult males (Figure 2B), (2) in juveniles: vertical bars are distinct and extend to the end of the ventral abdomen (Figure 2C). In adult males: vertical bars gradually vanish with increasing age. In adult females: vertical bars gradually recede above the lateral line with increasing age. (3) The last unbranched dorsal-fin ray is slender, and the posterior margin smooth. (4) Intestinal coiling is folded and elongated (Figure 2D,E).

**Description.** (Figure 2) The body is elongated and laterally compressed. The dorsal body profile rises from the nape to the origin of the dorsal-fin, gradually lowering to the caudal-fin base. The ventral body profile is round. The maximum body depth is at the origin of the dorsal-fin, and the body depth is 22.9–27.3% of the SL. The head is moderately large, and the length is almost equal to the body depth. The snout is blunt, and the length is 36.2–40.6% of the HL; two pairs of nostrils are positioned closer to the anterior margin of the orbit than to the snout tip; the eye is moderately, dorsolaterally positioned, and the diameter is 20.4–26.7% of the head length. The interorbital region is broad and convex, and the width is 129.6–198.4% of the eye diameter. The anus is closely positioned anterior to the origin of the anal-fin. The caudal peduncle is robust, laterally compressed, with a length of 15.1–17.9% of the SL and a depth of 34.2–68.2% of the caudal peduncle length (Table 1).

**Figure 3:**
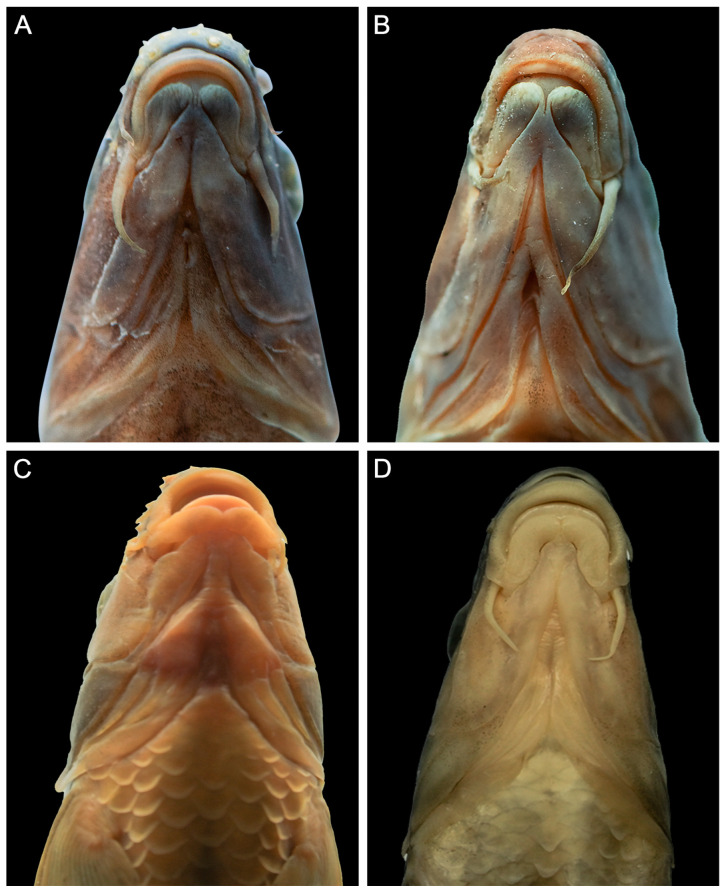
Comparisons of oral lips of *Acrossocheilus dabieensis* **sp. nov.**, *Acrossocheilus jishouensis*, and *Acrossocheilus kreyenbergii*. (**A**) Male adult of *A. dabieensis* **sp. nov.**, ventral view (HENUJGT002); (**B**) female adult of *A. dabieensis* **sp. nov.**, ventral view (HENUJGT001); (**C**) male adult of *A. jishouensis*, ventral view (ASIZB184956); and (**D**) male adult of *A. kreyenbergii*, ventral view (ASIZB223833).

The mouth is horse-shoe shaped and subterminal. There are two pairs of barbels, and the rostral barbel extends over the maxillary barbel base with the maxillary barbel extending under the midpoint of the eye. The mouth width is one-third of the head width. The lips are thick, and the upper lip is complete, connected with the lower lip at the corners of the mouth, with a groove between the rostral fold and upper jaw. The lower lip is well-developed, bisected into two lobes with a longitudinal shallow groove it the middle, completely separated from the lower jaw by a groove along the anterior profile of the lower lip. The lower jaw is exposed anterior to the lower lip, with a horny margin (Figure 3A,B).

The body is covered with moderately small cycloid scales. The lateral line is complete, slightly bending down below the origin of the dorsal-fin, then extending straight to the caudal-fin base. The lateral line scales total 38 (66 specimens) and 39 (27); the scales above the lateral line total 5.5 (93); the scales below the lateral line total 3 (93); and the pre-dorsal scales total 11 (58), 12 (35). There are 16 circumpeduncular scales (93).

**Figure 4:**
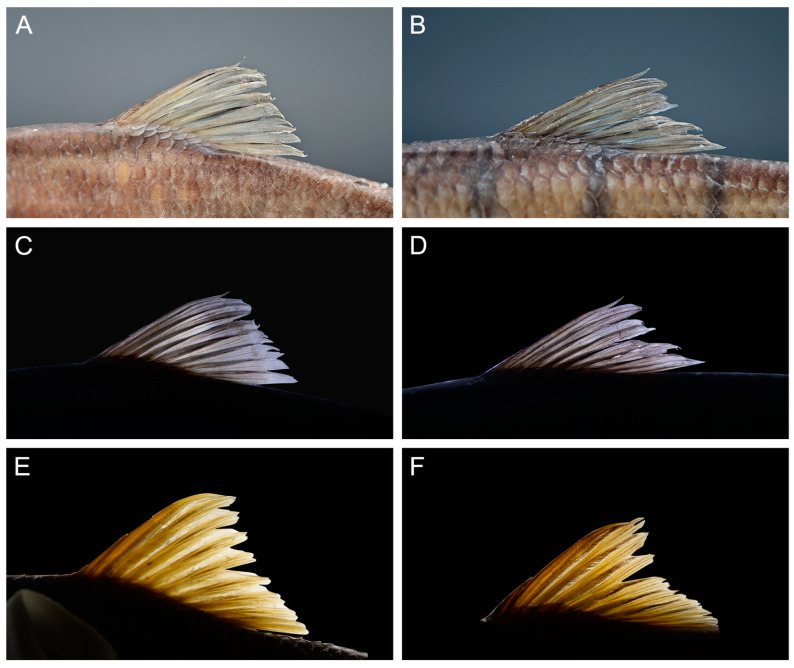
Comparisons of dorsal fins of *Acrossocheilus dabieensis* **sp. nov.** and *Acrossocheilus kreyenbergii*. (**A**) Male adult of *A. dabieensis* **sp. nov**., lateral view (HENUJGT002); (**B**) female adult *A. dabieensis* **sp. nov.**, lateral view (HENUJGT001); (**C**) male adult of *A. dabieensis* **sp. nov.**, transparent view; (**D**) female adult of *A. dabieensis* **sp. nov.**, transparent view (HENUJGT001); (**E**) male adult of *A. kreyenbergii*, transparent view (ASIZB223833); and (**F**) female adult of *A. kreyenbergii*, transparent view (ASIZB231514).

The dorsal fin has three unbranched and eight (93 specimens) branched rays; the last unbranched ray is slender and has a posterior margin without serration (Figure 4). The dorsal fin is shorter than the head length, with the origin equidistant from the snout tip and caudal-fin base. The pectoral fin has one unbranched and 12 (93) branched rays, and the tip of the depressed fin does not reach the pelvic-fin insertion. The pelvic fin with one unbranched and eight (92) branched rays is inserted below the first branched dorsal-fin ray; the tip of the depressed fin extends midway to the origin of the anal-fin. The anal fin with three unbranched and five (93) branched rays has an origin equidistant between the pelvic-fin insertion and the caudal-fin base. The caudal fin is deeply forked, with one unbranched principal ray and nine branched principal rays on the upper lobes, eight branched rays and one unbranched principal ray on the lower lobes (93) with pointed lobes. Gill rakers total 14 (17), 15 (53) and 16 (23) and are abbreviated and pointed. There is an air bladder in two chambers, and the anterior chamber is oval-shaped. The posterior chamber is elliptical with a length twice that of the anterior chamber. The intestine complex has six turns, and the intestinal length is approximately 1.85 ± 0.1 of the SL (Figure 2D,E).

**Coloration of fresh specimen.** Under natural conditions or immediately after collection, the head, dorsum, and upper sides are dark brown to black, while the lower sides are dark purple. Approximately two-thirds of the dorsal-fin membrane is black. The bases of the dorsal, pectoral, pelvic, anal, and caudal fins display a coloration from dark brown to black, with a prominent orange–red posterior margin. In a laboratory setting, with enhanced light intensity, the following results are found: (1) Adult females: The head, dorsum, and sides become yellowish-brown, and the vertical bars are more prominent. The dorsal, pectoral, pelvic, anal, and caudal fins are yellowish-brown with a light orange–red margin. (2) Adult males: The head, dorsum, and upper sides are yellowish-brown, with the black pigmentation in the dorsal-fin membrane being more accentuated. The dorsal, pectoral, and anal fins appear yellowish-brown and have a transparent appearance, with an orange –yellow margin. The pelvic fins are strikingly orange–red. (3) Juveniles: The head, dorsum, and sides are light yellowish-brown, with prominent vertical bars running along the sides. The dorsal, pectoral, pelvic, anal, and caudal fins are all light yellowish-brown.

**Coloration in preservation.** (Figure 2) The dorsal and lateral body is brown, and the ventral side is brownish-yellow in adults. There are six black vertical bars on the flank. The first vertical bar is above the pectoral fin. The second vertical bar is slightly anterior to the origin of the dorsal-fin. The third vertical bar is below the dorsal-fin base end. The fourth vertical bar is posterior to tip of the depressed pectoral fin. The fifth vertical bar is above the anal-fin base end. The sixth vertical baron is on the caudal-fin base. It extends vertically through the dorsal and ventral origins of the caudal fin. The black bars are about two or three scales in width. In juveniles, the black vertical bars extend beyond the lateral line. They extend somewhat towards the abdomen. In female adults, the black vertical bars are restricted above the lateral line. Longitudinal stripes are only present in male adults, extending along the lateral line. The dorsal-fin membrane is black pigmented, and the distal margin is transparent. The pectoral, ventral, anal and caudal-fin membranes lack black pigmentation and are grayish-yellow.

**Sexual dimorphism.** Tubercles on the snout are more significant in males than in females. The upper lip is more fleshy in females. The black vertical bars in adult males are faded or absent, while they exist in adult females. Adult females possess a slightly longer anal fin than adult males. The male abdomen turns light red in the breeding season. Vertical bars extend to the end of the ventral abdomen in juveniles, gradually receding above the lateral line in adult females, whereas they are absent in adult males.

**Figure 5:**
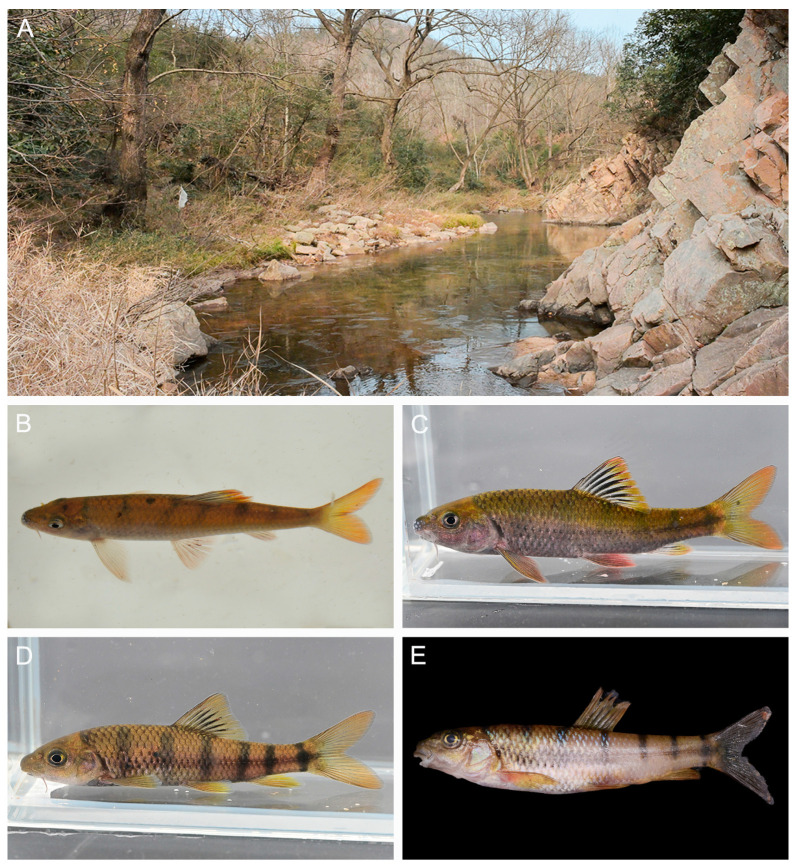
Habitat and adult habitus of *Acrossocheilus dabieensis* **sp. nov.** (**A**) Type locality in Liankangshan National Nature Reserve, Luoshan County, Henan Province, China; (**B**) male subadult habitus (HENUJGT0019); (**C**) male adult habitus (HENUJGT0010); and (**D**) female subadult habitus (HENUJGT0011); (**E**) female adult habitus (HENULKS0012).

**Distribution and Ecology.** Based on our collection records, *Acrossocheilus dabieensis* **sp. nov.** is known only from the Huaihe River Basin, Dabie Mountains, Henan Province, China. The new species mainly inhabits clear ravine streams and deep pools in this mountainous region (Figure 5A).

**Etymology.** The name of the new species, *dabieensis*, is derived from the name of the mountains, Dabie Mountains, the type locality of the new species (Figure 1). The suggested common English name is “Dabie Mountains *Acrossocheilus*” and the Chinese name is “Dà Bié Shān Guāng Chún Yú”.

### 3.2. Phylogenetic Analyses

**Figure 6:**
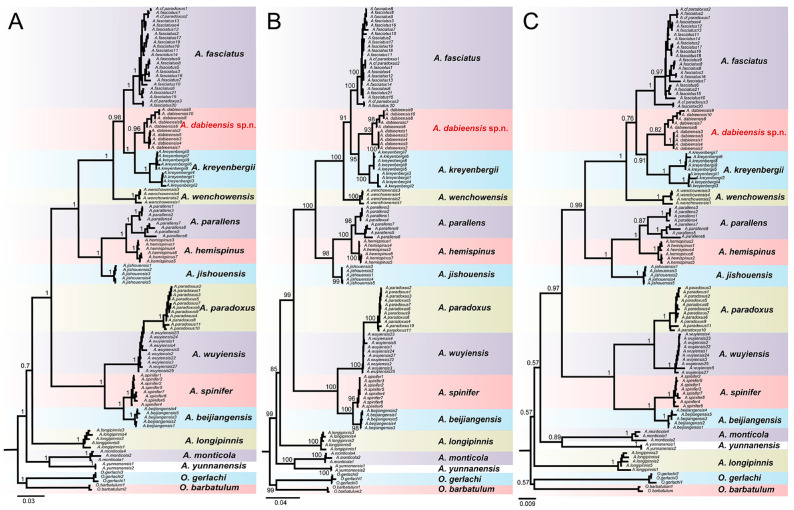
Phylogenetic trees of the genus *Acrossocheilus* based on the mitochondrial D-loop region genes. (**A**) Bayesian inference (BI) tree, numerals at nodes are Bayesian posterior probabilities; (**B**) Maximum likelihood (ML) tree, numerals at nodes are bootstrap values; (**C**) Neighbor-Joining (NJ) tree, numerals at nodes are bootstrap values.

A total of 122 D-Loop gene sequences were successfully obtained for the 14 species of *Acrossocheilus*. After alignment and terminal trimming, a total of 852 base pairs were employed to reconstruct phylogenetic trees. The D-Loop gene sequences exhibit pronounced A-T preferences (66.8%), in accordance with the situation of other fish. The BI, ML, and NJ trees (Figure 6) yielded similar topologies to the species of *Acrossocheilus* except for the collapsed relationship between *A. paradouxus* and *A. wuyiensis*. Individuals of *Acrossocheilus dabieensis* **sp. nov.** are confirmed to form a monophyletic clade with relatively high support values. *Acrossocheilus dabieensis* **sp. nov.** forms a sister taxon to *A. kreyenbergii* with high support values, suggesting a good species that separated from *A. kreyenbergii* and *A. jishouensis*.

## 4. Discussion

*Acrossocheilus dabieensis* **sp. nov.** differs from other species of the barred group (*A. beijiangensis, A. clivosius, A. fasciatus, A. hemispinus, A. iridescens, A. jishouensis, A. kreyenbergii, A. longipinnis, A. microstoma, A. monticola, A. paradoxus, A. parallens, A. spinifer, A. wenchowensis*, and *A. wuyiensis*) based on the following combination of characters: vertical stripes extending to the abdomen in juveniles (Figure 2C); in subadult females, vertical stripes gradually receding toward the lateral line, whereas they are confined above the lateral line in adult females (Figure 5D,E); in subadult males, vertical stripes beginning to appear as indistinct and only being found above the lateral line, whereas they are absent in adult males (Figure 5B,C); the PB2 being situated beneath the anterior origin of the dorsal-fin in females and subadult males; the last unbranched dorsal-fin ray being slender with a smooth posterior margin (Figure 3A–D); and intestinal coiling being folded with six turns (Figure 2D,E).

*Acrossocheilus dabieensis* **sp. nov.** can be discriminated from *A. beijiangensis*, *A. iridescens*, *A. longipinnis*, *A. monticola*, *A. paradoxus*, *A. spinifer*, and *A. wuyiensis* by the position of the PB2 on the flanks [25]. In the new species, the PB2 is located below the anterior origin of the dorsal fin (Figure 2B,C and Figure 5B,D,E), whereas in *A. beijiangensis*, *A. iridescens*, *A. longipinnis*, *A. monticola*, *A. paradoxus*, *A. spinifer*, and *A. wuyiensis*, the PB2 is situated below the posterior origins of the dorsal fin [2,6,11]. The new species shares a consistent trait with *A. fasciatus*, *A. kreyenbergii*, *A. jishouensis*, *A. parallens*, *A. hemispinus*, and *A. wenchowensis* in the position of PB2, which is located below the anterior origin of the dorsal fin [6,12].

*Acrossocheilus dabieensis* **sp. nov.** can be differentiated from *A. bejiangensis*, *A. paradoxus*, *A. spinifer*, *A. wenzhouensis*, and *A. fasciatus* by the extension of vertical stripes on the flanks. Vertical stripes are confined above the lateral line in adult females (Figure 2B and Figure 5E), and they are absent in adult males (Figure 5B,C). In adults of *A. paradoxus*, *A. bejiangensis* and *A. spinifer*, vertical stripes extend below the lateral line by 1–2 rows of scales [6]. In *A. wenzhouensis* and *A. fasciatus*, vertical stripes of adult females extend downward beyond the lateral line by three rows of scales, whereas vertical stripes are only confined above the lateral line in adult males [6].

*Acrossocheilus dabieensis* **sp. nov.** can be distinguished from *A. kreyenbergii*, *A. hemispinus*, and *A. parallens* by the robustness of the last unbranched dorsal-fin ray and the presence or absence of serrations on its posterior margin (Figure 4). Specifically speaking, in *A. dabieensis* **sp. nov.**, the last unbranched dorsal-fin ray is slender and delicate, with a smooth posterior margin lacking serrations (Figure 4A–D). In contrast, *A. kreyenbergii* (Figure 4D,E), *A. hemispinus*, and *A. parallens* have a robust last unbranched dorsal-fin ray with distinct fine serrations on the posterior margin.

*Acrossocheilus dabieensis* **sp. nov.** can be separated from *A. jishouensis* by the length of the postlabial barbel, the length of the rostral barbel, and the intestinal coiling pattern. The postlabial barbel of the new species is long and extends to the midpoint below the eye diameter (cf., it is short and reaches at the lower edge of the eye) (Figure 3A–C). The rostral barbel of the new species is short (36.2–38.3% of the HL) and does not reach the anterior margin of the eye (cf., it extends beyond the anterior margin of the eye) (Figure 3A–C). Furthermore, the intestinal coiling pattern of the new species is complex with six turns, and the intestinal length is approximately 1.85 times of the SL (Figure 2D,E), whereas the intestine of *A. jishouensis* is short and simple, only with two turns [34]. Additionally, phylogenetic analyses indicate that the new species is distantly related to *A. jishouensis* (Figure 6). These differences can distinguish *A. dabieensis* **sp. nov.** from *A. jishouensis*.

Specimens of *A. dabieensis* **sp. nov.** collected from the Dabie Mountains in Henan were initially identified as *A. kreyenbergii* in a previous study [18]. However, the last unbranched dorsal-fin ray of the new species is slender and fragile, with a smooth posterior margin, a characteristic that readily distinguished it from *A. kreyenbergii*. Initially, we speculated that these specimens had not reached sexual maturity, which could potentially explain the aforementioned trait [19,20]. Nevertheless, specimens collected from 2017 to 2025 refuted this hypothesis: the last unbranched dorsal-fin ray in sexually mature specimens remained smooth. Consequently, the species of *Acrossocheilus* in this region is not *A. kreyenbergii*, but rather a distinct new species, hereby described as *A. dabieensis* **sp. nov.**

## 5. Conclusions

A new species of the cyprinid genus *Acrossocheilus* Oshima, 1919, *A. dabieensis* **sp. nov.** is illustrated and described based on phylogenetic and morphological evidence. The new species is known only from the ravine streams of the Dabie Mountains, which is the northernmost boundary of the generic distribution range. The discovery of the new species not only enriches our understanding of fish diversity but also highlights the importance of the Dabie Mountains as a biodiversity hotspot in central China.

Key to the barred species of Acrossocheilus*

1a. Mouth opening horse-shoe shaped; lower lip covered by soft tissue
*A. iridescens* (Nichols & Pope, 1927)1b. Mouth opening arc-shaped; lower lip with a naked lower jaw22a. Mouth small, body laterally with dark spots
*A. microstoma* (Pellegrin & Chevey,1936)2b. Mouth large, body laterally with vertical stripes33a. Gill cover posterosuperiorly with a crescent-shaped purplish-black blotch
*A. monticola* (Giinther,1888)3b. Gill cover posterosuperiorly without crescent-shaped purplish-black blotch
44a. Vertical stripes yellowish-brown*A. longipinnis* (Wu, 1939)4b. Vertical stripes black55a. Vertical bars not stable or without regular variation, varying in pattern, number, width, and position of body; supracleithrum ventrally without protuberance
*A. clivosius* (Linnaeus, 1935)5b. Vertical bars stable or with regular variation in pattern, number, width, and position; supracleithrum ventrally with protuberance66a. Dorsal-fin membrane transparent76b. Dorsal-fin membrane not transparent87a. Rostral barbel reaches the anterior margin of the eye
*A. parallens* (Nichols, 1931)7b. Rostral barbel does not reach the anterior margin of the eye
*A. hemispinus* (Nichols, 1925)8a. PB2 posterior to the origin of dorsal-fin98b. PB2 anterior to the origin of dorsal-fin129a. Lower lip lobes closely appressed with a narrow gap; dorsal-fin membrane partially black109b. Lower lip lobes either closely appressed or distinctly separated; dorsal-fin membrane absolutely black1110a. Width of vertical bars spanning 3–4 scales
*A. beijiangensis* Wu & Lin, 197710b. Width of vertical bars spanning 2 scales
*A. spinifer* Yuan, Wu & Zhang, 200611a. Posterior margin of last unbranched dorsal-fin ray smooth, without serrations
*A. paradoxus* (Gunther, 1868)11b. Posterior margin of last unbranched dorsal-fin ray with fine serrations
*A. wuyiensis* Wu & Li, 198112a. Adult males with vertical bars1312b. Adult males without vertical bars1413a. Width of vertical bars spanning 1 scale in adults
*A. wenchowensis* Wang, 193513b. The length of rostral barbel does not reach anterior margin of eye in adults
*A. fasciatus* (Steindachner, 1892)14a. Last unbranched dorsal-fin ray robust, with fine serrations on posterior margin
*A. kreyenbergii* (Regan, 1908)14b. Last unbranched dorsal-fin ray slender, without serrations on posterior margin
1515a. Maxillary barbel reaches anterior margin of eye; intestinal coiling pattern simple, with 2 turns*A. jishouensis* Zhao, Chen & Li, 199715b. Maxillary barbel does not reach anterior margin of eye; intestinal coiling long and complex, with 6 turns*A. dabieensis* **sp. nov.**

* *Acrossocheilus lamus* (Mai, 1978) is not included in the key because of the lack of informative characters [25].

**Comparative Materials:**

*Acrossocheilus beijiangensis*

ASIZB 63891–63892, two specimens, 95.6–128.6 mm SL; Longsheng Village, Rongan City, Guangxi Autonomous Region, China; 1974.

*Acrossocheilus spinifer*

ASIZB 228566, one specimen, 38.4 mm SL; X809 Rode, Jianyang County town, Nanping City, Fujian Province, China; 17 April 2021.

*Acrossocheilus wuyiensis*

ASIZB 228298–228300, three specimens, 99.8–107.3 mm SL, Guangze County town, Nanping City, Fujian Province, China; 17 April 2021. ASIZB 226943, 226946–226947, three specimens, 85.1–95.2 mm SL, Gaosuban Market, Qishan Industrial Park, Wuyishan City, Fujian Province, China; 13 April 2021. ASIZB 227079, one specimen, 70.3 mm SL, G237 water tank, Wuyishan City, Fujian Province, China; 12 April 2021. ASIZB 230531, 230532, two specimens, 95.2–97.8 mm SL, Qishanta Village, Wuyishan City, Fujian Province, China; 18 July 2021. ASIZB 230684, 230698, two specimens, 37.8–70.1 mm SL, Wuyishan Nature Reserve Rare Plants Garden, Wuyishan City, Fujian Province, China; 18 July 2021.

*Acrossocheilus wenchowensis*

ASIZB 228967, 228968, two specimens, 46.3–64.9 mm SL, Hongan Village, Jinyun County town, Lishui City, Zhejiang Province, China; 19 April 2021. ASIZB 228923, 228927, 228928, three specimens, 59.8–96.8 mm SL, Yazhai Village, Jinyun County town, Lishui City, Zhejiang Province, China; 19 April 2021. ASIZB 229061, 229062, two specimens, 78.8–84.9 mm SL, Xiandu National Scenic Area, Lishui City, Zhejiang Province, China; 20 April 2021. ASIZB 228795, one specimens 97.9 mm SL, Guyangtou Village, Yunhe County town, Lishui City, Zhejiang Province, China; 20 April 2021. ASIZB 228658, 228659, two specimens, 52.2–65.4 mm SL, Hegong bridge, Jianyang County town, Nanping City, Fujian Province, China; 17 April 2021.

*Acrossocheilus fasciatus*

ASIZB 126688, one specimen, 93.4 mm SL, Southern Anhui Province, China; 1961.

*Acrossocheilus kreyenbergii*

ASIZB 231512–231517, six specimens, 98.5–113.9 mm SL, Wusan Trade Market, Guangxin District, Shangrao City, Jiangxi Province, China; 21 July 2021. ASIZB 229243–229244, two specimens, 66.4–72.2 mm SL, Binjiang Road East Section, Wuyuan County town, Shangrao City, Jiangxi Province, China; April 2021. ASIZB 22945, one specimen, 50.6 mm SL, Qianshan County town, Shangrao City, Jiangxi Province, China; 19 April 2021.

*Acrossocheilus jishouensis*

ASIZB 184941, 184956, two specimens, 44.9–71.5 mm SL, Yuan River, Yangtze tributary, Chenxi County town, Hunan Province, China; 29 March 2002. ASIZB 184529–184531, three specimens, 84.2–114.5 mm SL, Yuan River, Yangtze tributary, Chenxi County town, Hunan Province, China; 7 April 2002. ASIZB 184648, 184650–184651, three specimens, 36.9–73.5 mm SL, Zishui River, Yangtze tributary, Gongqiao Village, Shaoyang City, Hunan Province, China; 29 March 2002.

*Acrossocheilus hemispinus*

ASIZB 231173, 231174, two specimens, 116.9–122.4 mm SL, Route 217, Guangzi County town, Nanping City, Fujian Province, China; 13 April 2021. ASIZB 231295, 231301, two specimens, 125.9–126.5 mm SL, Qishanta Village, Wuyishan City, Fujian Province, China; 18 July 2021. ASIZB 230882, 230892, two specimens, 50.3–67.3 mm SL, Yunhe County town, Lishui City, Zhejiang Province, China; 20 April 2021. ASIZB 230778, one specimen 92.6 mm SL, Jianyang County town, Nanping City, Fujian Province, China; 13 April 2021. ASIZB 199076, one specimen, 109.3 mm SL, Xingan County town, Zhuang Autonomous Prefecture, Guangxi Autonomous Region, China; 23 September 1984.

*Acrossocheilus parallens*

ASIZB 184387, 184389, two specimens, 59.2–65.2 mm SL, Meizhong Village, Butou County town, Hezhou City, Zhuang Autonomous Prefecture, Guangxi Autonomous Region, China; 27 March 2003. ASIZB 62516, one specimen, 109.3 mm SL, Jian City, Jiangxi Province, China; 27 March 2003. ASIZB 183808, 183810, two specimens, 63.1–82.9 mm SL, Xijiang tributary, Li River, Lingchuan County town, Guilin City, Zhuang Autonomous Prefecture, Guangxi Autonomous Region, China; 26 March 2003.

## Figures and Tables

**Table 1:** Morphometric measurements of *Acrossocheilus dabieensis* **sp. nov.**

Character	Holotype	Paratypes (*n* = 92)		
		Range	Mode	Mean
Dorsal-fin rays	iii-8	iii-8	iii-8	
Pectoral-fin rays	i-12	i-12	i-12	
Pelvic-fin rays	i-8	i-8	i-8	
Anal-fin rays	i-5	i-5	i-5	
Gill rakers	6 + 9 = 15	14–16	6 + 9 = 15	
Lateral line scales	38	38–40	38	
Standard length (mm)	100.2	28.6–121.2		88.9
In percentage of SL				
Head length	26.4	25.8–28.3		27.6
Body depth	26.3	21.3–22.9		24.1
Head height	19.8	17.7–24.8		18.8
Caudal peduncle length	15.1	17.9–32.1		17.4
Caudal peduncle depth	10.3	9.2–9.8		10.2
Predorsal length	53	46.5–51.3		51.6
Prepectoral length	23	25.6–27.3		26.2
Prepelvic-fin length	51.8	50.3–51.7		55.4
Preanal-fin length	72.3	75.7–78.0		77.3
Interorbital width	11.1	10.5–11.2		10.8
Pectoral-fin length	25.1	20.4–23.8		20.9
Anal-fin length	19.7	19.7–19.9		19.8
Dorsal-fin length	21.3	19.9–25.9		20.5
Pelvic-fin length	21.1	17.2–21.3		17.7
In percentage of HL				
Snout length	36.2	38.2–38.3		38.2
Eye diameter	21.9	20.4–27.2		24.6

## Data Availability

The data presented in this study are available on request from the corresponding author.

## Supplementary Materials

The following supporting information can be downloaded at: https://www.mdpi.com/article/10.3390/ani15050734/s1, Table S1: The species, drainages, collection sites, number of specimens, and GenBank accession numbers of the specimens for this study.
