# Supplementary material for: A New Species of the Genus Acrossocheilus Oshima, 1919 (Cypriniformes: Cyprinidae) from the Dabie Mountains
Source: Animals (Basel). 2025 Mar 4;15(5):734. doi: 10.3390/ani15050734 (PMC11899002; doi:10.3390/ani15050734)
Supplement: Supplementary file 1 [file animals-15-00734-s001.zip › animals-3470594-supplementary.pdf]

SUPPORTING MATERIAL

A New Species of the Genus *Acrossocheilus* Oshima, 1919 (Cypriniformes: Cyprinidae) from the Dabie Mountains

Table S1. Species, drainages, collection sites, number of specimens and GenBank accession numbers of specimens for this study.

Species	GenBank accession number	Specimens number	Localities
A.dabieensis 1	PQ249124	HENUJGT02	Xinyang, Henan
A.dabieensis 2	PQ249125	HENUJGT05	Xinyang, Henan
A.dabieensis 3	PQ249126	HENUJGT06	Xinyang, Henan
A.dabieensis 4	PQ249127	HENUJGT07	Xinyang, Henan
A.dabieensis 5	PQ249128	HENUJGT08	Xinyang, Henan
A.dabieensis 6	PQ249129	HENULKS05	Xinyang, Henan
A.dabieensis 7	PQ249130	HENULKS07	Xinyang, Henan
A.dabieensis 8	PQ249131	HENULKS10	Xinyang, Henan
A.dabieensis 9	PQ249132	HENULKS11	Xinyang, Henan
A.dabieensis 10	PQ249133	HENULKS12	Xinyang, Henan
A. beijiangensis 1	KT877111	IHB 2008090024	Lianzhou, Guangdong
A. beijiangensis 2	KT877109	IHB 2008090023	Lianzhou, Guangdong
A. beijiangensis 3	KT877112	IHB 2008090028	Lianzhou, Guangdong
A. beijiangensis 4	KT877108	IHB 2008090025	Lianzhou, Guangdong
A. beijiangensis 5	KT877110	IHB 2008090022	Lianzhou, Guangdong
A. cf. paradoxus 1	KT877040	IHB 2006052200	Linhai, Zhejiang
A. cf. paradoxus 2	KT877041	IHB 2006052214	Tiantai, Zhejiang
A. cf. paradoxus 3	KT877057	IHB 2006052223	Tiantai, Zhejiang
A. fasciatus 9	KT877056	IHB 2003050003	Huangshan, Anhui
A. fasciatus 10	KT877060	IHB 2008090049	Huangshan, Anhui
A. fasciatus 11	KT877045	IHB 2006052275	Huangshan, Anhui
A. fasciatus 12	KT877061	IHB 2008090051	Huangshan, Anhui
A. fasciatus 13	KT877043	IHB 2003050004	Huangshan, Anhui
A. fasciatus 14	KT877042	IHB 2003050002	Huangshan, Anhui
A. fasciatus 15	KT877052	IHB 2007100006	Huangshan, Anhui
A. fasciatus 16	KT877054	IHB 2006052244	Xianju, Zhejiang
A. fasciatus 17	KT877049	IHB 2006052251	Xianju, Zhejiang
A. fasciatus 18	KT877048	IHB 2006052268	Xianju, Zhejiang

A. fasciatus 19	KT877047	IHB 2006052247	Xianju, Zhejiang
A. fasciatus 20	KT877058	IHB 2006052269	Xianju, Zhejiang
A. fasciatus 21	KT877051	IHB 2006052201	Jiande, Zhejiang
A. hemispinus 1	KT877074	IHB 2007050018	Yongtai, Fujian
A. hemispinus 3	KT877073	IHB 2007050017	Yongtai, Fujian
A. hemispinus 4	KT877075	IHB 2006052204	Nanping, Fujian
A. hemispinus 5	KT877076	IHB 2007050007	Nanping, Fujian
A. hemispinus 6	KT877077	IHB 2007050015	Nanping, Fujian
A. hemispinus 7	KT877078	IHB 2007050014	Nanping, Fujian
A. jishouensis 1	KT877088	IHB 2008050035	Huaihua, Hunan
A. jishouensis 2	KT877091	IHB 2008050038	Huaihua, Hunan
A. jishouensis 3	KT877090	IHB 2008050036	Huaihua, Hunan
A. jishouensis 4	KT877089	IHB 2008050039	Huaihua, Hunan
A. jishouensis 5	KT877092	IHB 2008050037	Huaihua, Hunan
A. kreyenbergii 1	KT877065	IIHB 2006052286	Yiyang, Jiangxi
A. kreyenbergii 2	KT877066	IHB 2006052282	Yiyang, Jiangxi
A. kreyenbergii 3	KT877063	IHB 2006052285	Yiyang, Jiangxi
A. kreyenbergii 4	KT877064	IHB 2006052284	Yiyang, Jiangxi
A. kreyenbergii 5	KT877067	IHB 2006052238	Rongshui, Guangxi
A. kreyenbergii 6	KT877072	IHB 2006052254	Rongshui, Guangxi
A. kreyenbergii 7	KT877070	IHB 2006052243	Rongshui, Guangxi
A. kreyenbergii 8	KT877071	IHB 2004090007	Rongshui, Guangxi
A. kreyenbergii 9	KT877068	IHB 2004090006	Rongshui, Guangxi
A. kreyenbergii 10	KT877069	IHB 2004090010	Rongshui, Guangxi
A. paradoxus 2	AJ274665	Unknown	Houlong, Taiwan
A. paradoxus 3	AJ274664	Unknown	Houlong, Taiwan
A. paradoxus 4	AJ274678	Unknown	Houlong, Taiwan
A. paradoxus 5	AJ131834	Unknown	Houlong, Taiwan
A. paradoxus 6	AJ274670	Unknown	Houlong, Taiwan
A. paradoxus 7	AJ274666	Unknown	Houlong, Taiwan
A. paradoxus 8	AJ274663	Unknown	Houlong, TTaiwan
A. paradoxus 9	AJ274667	Unknown	Houlong, Taiwan
A. paradoxus 10	AJ300613	Unknown	Chuoshui, Taiwan
A. paradoxus 11	AJ132080	Unknown	Houlong, Taiwan
A. wuyiensis 1	KT877094	IHB 20070600053	Shaowu, Fujian
A. wuyiensis 2	KT877098	IHB 20070600078	Jianyang, Fujian
A. wuyiensis 3	KT877095	IHB 20070600081	Jianyang, Fujian
A. wuyiensis 4	KT877096	IHB 20070600005	Jian’ou, Fujian
A. wuyiensis 5	KT877097	IHB 0070600004	Jian’ou, Fujian

A. wuyiensis 22	KT877102	IHB 20070600001	Jian'ou, Fujian
A. wuyiensis 23	KT877099	IHB 20070600012	Jian'ou, Fujian
A. wuyiensis 24	KT877093	IHB 20070500059	Wuyishan, Fujian
A. wuyiensis 25	KT877100	IHB 20070500060	Wuyishan, Fujian
A. wuyiensis 26	KT877101	IHB 20070500064	Wuyishan, Fujian
A. wuyiensis 27	KT877103	IHB 20070500065	Wuyishan, Fujian
A. kreyenbergii 1	KT877065	IIHB 2006052286	Yiyang, Jiangxi
A. kreyenbergii 2	KT877066	IHB 2006052282	Yiyang, Jiangxi
A. kreyenbergii 3	KT877063	IHB 2006052285	Yiyang, Jiangxi
A. kreyenbergii 4	KT877064	IHB 2006052284	Yiyang, Jiangxi
A. kreyenbergii 5	KT877067	IHB 2006052238	Rongshui, Guangxi
A. kreyenbergii 6	KT877072	IHB 2006052254	Rongshui, Guangxi
A. kreyenbergii 7	KT877070	IHB 2006052243	Rongshui, Guangxi
A. kreyenbergii 8	KT877071	IHB 2004090007	Rongshui, Guangxi
A. kreyenbergii 9	KT877068	IHB 2004090006	Rongshui, Guangxi
A. kreyenbergii 10	KT877069	IHB 2004090010	Rongshui, Guangxi
A. longipinnis 1	KT877128	IHB 2008050040	Baise, Guangxi
A. longipinnis 2	KT877126	IHB 2008050043	Baise, Guangxi
A. longipinnis 3	KT877125	IHB 2008050041	Baise, Guangxi
A. longipinnis 4	KT877127	IHB 2008050042	Baise, Guangxi
A. longipinnis 5	KT877129	IHB 2004090011	Tian'e, Guangxi
A. monticola 1	KT877123	IHB 2004090003	Hechuan, Chongqing
A. monticola 2	KT877124	IHB 2004090001	Hechuan, Chongqing
A. monticola 4	KT877122	IHB 2004090002	Hechuan, Chongqing
A. parallens 1	KT877080	IHB 2005050003	Guanshan, Jiangxi
A. parallens 2	KT877082	IHB2005050004	Guanshan, Jiangxi
A. parallens 3	KT877081	IHB 2005050002	Guanshan, Jiangxi
A. parallens 4	KT877083	IHB 2005050002	Guanshan, Jiangxi
A. parallens 5	KT877084	IHB 2008090026	Lianzhou, Guangdong
A. parallens 6	KT877087	IHB 2008090030	Lianzhou, Guangdong
A. parallens 7	KT877085	IHB 2008090027	Lianzhou, Guangdong
A. parallens 8	KT877086	IHB 2008090029	Lianzhou, Guangdong
A. spinifer 1	KT877104	IHB 2007050008	Nanping, Fujian
A. spinifer 2	KT877105	IHB 2007050016	Nanping, Fujian
A. spinifer 3	KT877107	IHB 2007050012	Nanping, Fujian
A. spinifer 4	KT877106	IHB 2007050013	Nanping, Fujian
A. spinifer 5	KT877114	IHB 2008090034	Shanghang, Fujian
A. spinifer 6	KT877116	IHB 2008090031	Shanghang, Fujian
A. spinifer 7	KT877113	IHB 2008090032	Shanghang, Fujian
A. spinifer 8	KT877115	IHB 2008090033	Shanghang, Fujian

A. wenchowensis 1	KT877038	IHB 20060502211	Lishui, Zhejiang
A. wenchowensis 2	KT877037	IHB 20060502209	Lishui, Zhejiang
A. wenchowensis 3	KT877035	IHB 20060502207	Lishui, Zhejiang
A. wenchowensis 4	KT877036	IHB 20060502208	Lishui, Zhejiang
A. yunnanensis 1	KT877119	IHB 0403096	Chishui, Guangzhou
A. yunnanensis 2	KT877120	IHB 0403097	Chishui, Guangzho
O. barbatula 1	KT877117	IHB 2006050055	Shaowu, Fujian
O. barbatula 2	KT877118	IHB 2006050056	Shaowu, Fujian
O. gerlachi 1	KT877132	IHB 2008090019	Unknown
O. gerlachi 2	KT877130	IHB 2008090020	Unknown
O. gerlachi 3	KT877131	IHB 2008090021	Unknown
